# Spatiotemporal dynamics of soil bacterial and fungal communities at a small scale

**DOI:** 10.3389/fmicb.2026.1924980

**Published:** 2026-09-11

**Authors:** Hongling Zhang, Qianchong Ran, Qin Sun, Qi Lu, Qiyan Wang, Yi Tang, Bin Li, Chi Zhang, Zheng Ren, Xiaoye Jin

**Affiliations:** 1 Department of Forensic Medicine, Guizhou Medical University, Guiyang, China; 2 School of Criminal Science and Technology, People's Public Security University of China, Beijing, China; 3 Key Laboratory of Forensic Genetics of Ministry of Public Security, Institute of Forensic Science, Ministry of Public Security, Beijing, China

**Keywords:** bacteria, fungi, geographic provenance, soil, spatiotemporal dynamics

## Abstract

**Background:**

Soil is a common form of trace evidence in forensic investigations, and its indigenous microbial communities hold promise for geographic provenance determination. However, most existing studies focus on macro-scale soil microbial patterns, with limited understanding of their small-scale spatiotemporal dynamics. This study collected soil samples across multiple time points from a small-scale forest site in Guiyang, China, to characterize bacterial and fungal community distributions. We aimed to quantify the effects of temporal variation and geographic distance on soil bacterial and fungal community structure, and to evaluate their utility as biomarkers for geographic provenance inference.

**Methods:**

Forest surface soil samples were collected from five geographic locations at five time points, with paired environmental and meteorological data recorded *in situ*. High-throughput sequencing of the 16S ribosomal RNA gene and the internal transcribed spacer 2 region was performed on the MGI DNBSEQ-G99 platform. We integrated alpha and beta diversity analyses, distance-decay models, neutral community models, and random forest algorithms to systematically assess spatiotemporal associations of microbial community assembly and the performance of soil microbes for small-scale geographic provenance prediction.

**Results:**

Bacterial Pielou's evenness differed significantly across sampling locations, but alpha diversity of neither bacteria nor fungi varied significantly within the observation window of this study. Permutational multivariate analysis of variance revealed that geographic location was strongly correlated with community structure variation, with no significant temporal effect within the 14-day observation window. Fungal communities exhibited a stronger distance-decay relationship, lower fit to neutral community models, and lower dispersal rate than bacterial communities, indicating stronger deterministic environmental selection. Both bacterial and fungal communities demonstrated exceptional potential for closed-set recognition of predefined sites (accuracy > 92%).

**Conclusion:**

Soil microbial community structure remains relatively stable over the study period, with spatial heterogeneity far exceeding temporal variability. In small-scale forest habitats with relatively stable vegetation, fungal communities exhibit stronger spatial heterogeneity than bacteria, a pattern likely driven by their high specificity for the co-varying host plants and elevation gradients across the sampling sites. However, inferences across different vegetation types and longer temporal scales require further validation.

## 1 Introduction

Soil is an extremely common and crucial type of trace evidence at crime scenes. According to Locard's Exchange Principle, when suspects or vehicles are present at a crime scene, soil particles often adhere to their footwear, tires, or clothing. Comparative analysis of soil samples recovered from suspects or vehicles against those from suspected crime scenes can help determine potential incident locations and delineate suspect movement trajectories, thereby narrowing the investigation scope (Jurkevitch and Pasternak, 2021) and providing critical clues for case resolution. Traditional forensic soil examinations primarily rely on physicochemical properties such as color, particle size, and mineral composition; however, these methods offer limited resolution for tracing the geographic provenance of soil samples in highly homogeneous or complex terrains (Morgan and Bull, 2007). In recent years, with the advent of next-generation sequencing (NGS) technologies, soil microbes particularly bacteria and fungi have emerged as highly promising geographic fingerprints for characterizing soil provenance due to their high biodiversity and environmental specificity (Oliveira and Amorim, 2018).

Significant advances have been made in geographic provenance inference based on soil microbial communities. NGS technologies, such as 16S ribosomal RNA (16S rRNA) and internal transcribed spacer (ITS) targeted amplicon sequencing, have been widely applied to characterize microbial community profiles in environmental samples, and are increasingly integrated with machine learning algorithms to construct geographic prediction models. For example, Damaso et al. employed bioinformatics approaches to assess biogeographic patterns of soil communities across different spatial scales, demonstrating that the random forest (RF) algorithm achieved high classification accuracy for soil geographic provenance (Damaso et al., 2018). Similarly, Bhattacharya et al. confirmed that supervised machine learning methods can effectively extract geospatial association features from microbiomes, enabling high-precision geographic prediction at regional scales (Bhattacharya et al., 2022). Collectively, these studies indicated that soil microbial fingerprints possessed substantial potential for distinguishing macro-geographic regions. Nonetheless, several challenges remain unresolved in routine forensic practice. The first challenge concerns spatial micro-heterogeneity and biomarker selection. Even at the fine-scale spatial environment, microbial community structures may exhibit significant differentiation due to nutrient gradients or physical barriers (Ranjard and Richaume, 2001). Bacterial and fungal communities differ in their utility for geographic inference, and debate persists regarding which taxonomic group is more suitable as a biomarker for provenance discrimination (Young et al., 2014). Although bacterial communities exhibited high abundance and metabolic activity, their small cell size renders them highly susceptible to passive dispersal via aerosols or water currents; this enhanced dispersal capacity may compromise their resolution at local geographic scales (Joung et al., 2017). In contrast, mounting evidence suggested that fungal communities outperformed bacteria in distinguishing specific geographic locations (Efeoglu et al., 2022). Biologically, fungi are intimately associated with the soil matrix through extensive mycelial networks, and the dispersal of their relatively large spores depends heavily on specific biological or physical vectors, imposing a stronger dispersal limitation than that experienced by bacteria (Peay et al., 2016). Consequently, fungal communities are expected to more precisely reflect localized environmental filtering effects, thereby providing more specific geographic indicators for soil provenance in forensic applications. The second major challenge stems from temporal dynamics. In forensic practice, a time lag of several days to weeks often exists between the incident and evidence collection at the scene. A previous study has demonstrated that microbial community succession was jointly driven by deterministic processes (e.g., environmental selection) and stochastic processes (e.g., ecological drift over time) (Stegen et al., 2012). Microbial communities not only exhibited pronounced spatial heterogeneity in response to geographic location and local environmental conditions (Zhang et al., 2020), they were also affected by seasonal, climatic, and microenvironmental fluctuations; over time, these communities may undergo continuous succession and shifts in metabolic states (Docherty et al., 2015; Nguyen et al., 2021). A study on Norwegian soil samples further revealed that when forensic provenance models span different seasons or distinct time points, geographic prediction accuracy can decline sharply from over 85% to approximately 60% (Hofsvang et al., 2026). Therefore, evaluating the relative contributions of spatial heterogeneity and temporal variability to microbial community structure is a critical prerequisite for applying microbial biomarkers to soil geographic provenance.

Consequently, we investigated effects of geographic distances and temporal variation within an approximately 14-day observation window on soil bacterial and fungal community structures within a small-scale area. By integrating ecological statistical models and machine learning algorithms, we conducted an in-depth analysis of the spatial distribution patterns and environmental factors associated with bacterial and fungal communities, and evaluated the efficacy of bacterial and fungal communities as biomarkers for soil geographic provenance, with the aim of providing a theoretical basis and data support for forensic soil provenance inference.

## 2 Materials and methods

### 2.1 Sample collection

Forest surface soil (0–5 cm depth) was collected from Qianlingshan Park, Yunyan District, Guiyang City, Guizhou Province, China. Five sampling points (A–E) were established with successive pairwise distances of approximately 100, 200, 400, and 800 m (i.e., A–B, B–C, C–D, and D–E, respectively), with their detailed geographic coordinates listed in Table 1. Soil samples were collected on five dates: June 24, June 27, June 30, July 4, and July 8, 2025. At each sampling point on each date, three replicate composite samples were obtained. Briefly, within a 5 × 5 m area, three 1 × 1 m quadrats were randomly established. Within each quadrat, soil sample was collected by the five-point sampling method using a sterile soil auger and homogenized into a single composite sample, yielding a total of 75 samples (5 points × 5 dates × 3 replicates). Additionally, one blank swab tube was viewed as a field negative control at each site, totaling 25 negative control samples. In addition, altitude (AL), ambient temperature (Tm), and relative humidity (Hu) were recorded at each point using handheld GPS devices and portable thermo-hygrometers. Weather conditions (We) were documented qualitatively, and pairwise geographic distances (Di) between sampling points were derived from the GPS coordinates.

**Table 1:** The information of sampling sites.

sites	Latitude (*N*)	Longitude (*E*)	Elevation (*m*)	Dominant vegetation
A	26°36′6″	106°42′5″	1,117	Bamboo forest and shrubland
B	26°36′10″	106°42′5″	1,140	Bamboo-pine forest ecotone
C	26°36′12″	106°42′2″	1,174	Pine forest
D	26°36′12″	106°41′52″	1,190	Pine forest and shrubland
E	26°35′59″	106°41′34″	1,138	Pine forest and shrubland

The ‘Dominant Vegetation' column represents qualitative descriptions based on on-site photographic records, rather than quantitative land cover inventory data.

### 2.2 DNA extraction, polymerase chain reaction (PCR) amplification, and library construction

Total genomic DNA was extracted from soil samples using the Magnetic Bead-Based Soil DNA Extraction Kit (Fangzhou Biotechnology Co., Ltd., Chengdu, Sichuan, China), and their concentration and purity were assessed with a NanoDrop One spectrophotometer (Thermo Fisher Scientific, Waltham, MA, USA). The bacterial 16S rRNA gene V4 region and fungal ITS2 region were amplified using primer pairs 515F/806R (5′-GTGCCAGCMGCCGCGGTAA-3′ and 5′-GGACTACHVGGGTWTCTAAT-3′) and ITS3-F/ITS4-R (5′-GCATCGATGAAGAACGCAGC-3′ and 5′-TCCTCCGCTTATTGATATGC-3′), respectively. To monitor potential cross-contamination during amplification, sterile ultrapure water was used as a PCR negative control. All PCRs were performed on a Bio-Rad S1000 thermal cycler (Bio-Rad Laboratories, Inc., Hercules, CA, USA). For bacterial amplification, the 50 μL reaction mixture contained 25 μL of Premix Taq, 2.5 μL of each primer (10 μM), 50 ng of template DNA, and nuclease-free water. The thermal cycling conditions consisted of an initial denaturation at 94 °C for 5 min, followed by 31 cycles of denaturation at 94 °C for 30 s, annealing at 53 °C for 30 s, and extension at 72 °C for 30 s; with a final extension at 72 °C for 8 min, and held at 4 °C. For fungal amplification, the 50 μL reaction mixture contained 25 μL of 2 × Novoprotein Taq Master Mix, 2 μL of each primer (10 μM), 50 ng of template DNA, and nuclease-free water. The thermal cycling conditions consisted of an initial denaturation at 95 °C for 5 min, followed by 35 cycles of denaturation at 95 °C for 20 s, annealing at 56 °C for 20 s, and extension at 72 °C for 30 s; with a final extension at 72 °C for 5 min, and held at 4 °C. Following amplification, the amplicons were verified by 1.5% agarose gel electrophoresis, no amplification bands were observed in any of the negative control samples, confirming the absence of detectable contamination; thus, they were excluded from subsequent library construction and sequencing. DNA library was quantified using GeneTools (v4.03.05.0), pooled at equimolar ratios, and purified with the E.Z.N.A. Gel Extraction Kit (Omega Bio-tek, Norcross, GA, USA). Sequencing libraries were prepared using the ALFA-SEQ DNA Library Prep Kit (Fangzhou Biotechnology Co., Ltd., Chengdu, Sichuan, China), quality-checked on an Agilent 2100 Bioanalyzer (Agilent Technologies, Santa Clara, CA, USA), and sequenced on the MGI DNBSEQ-G99 platform.

### 2.3 Sequencing data processing

Raw sequencing reads were quality-filtered using fastp (v0.14.1) (Chen et al., 2018) with a 4-bp sliding window (mean quality threshold: Q20), and primer sequences were trimmed using cutadapt (v1.14) (Martin, 2011). Paired-end reads were merged using the fastq_mergepairs command in USEARCH (v10.0.240) with a minimum overlap of 16 bp and a maximum mismatch of 5 bp. Merged reads were quality-filtered again using fastp with the same sliding window parameters to generate high-quality clean reads. Sequence denoising and amplicon sequence variant (ASV) inference were performed using the DADA2 plugin in QIIME 2 (v2020.11.0) with default parameters to construct the ASV feature table. Following the generation of the ASV feature table, we strictly filtered out ASVs that had a total sequence count of only 1 across the global dataset. Following quality control, a total of 5,293,103 bacterial reads and 147,769 ASVs, alongside 5,276,925 fungal reads and 31,189 ASVs, were obtained from the 75 soil samples. Detailed sequencing statistics for each sample are provided in Supplementary Table 1. The data were rarefied and standardized to a uniform depth based on the minimum sequencing depth (42,020 for bacteria and 29,140 for fungi). Taxonomic annotation of bacterial ASVs was conducted against the SILVA database (v138) (Quast et al., 2013), and fungal ASVs against the UNITE database (v8.0) (Nilsson et al., 2019).

### 2.4 Statistical analysis

Alpha diversity metrics, including richness, Shannon index, and Pielou evenness, were computed using the vegan (v2.7-1) and phyloseq (v1.26.1) packages in R (v4.5.1). Inter-group differences in alpha diversity were evaluated via Kruskal–Wallis rank-sum tests, with *P*-values adjusted using the Benjamini–Hochberg correction for multiple comparisons. To characterize community structure, principal coordinate analysis (PCoA) based on Bray–Curtis dissimilarities was performed. The effects of spatiotemporal factors on community structure were quantified using permutational multivariate analysis of variance (PERMANOVA) with 999 permutations. To ensure that the observed differences were associated with spatial location rather than dispersion heterogeneity, tests of homogeneity of multivariate dispersions (betadisper) were also conducted. *P*-values for both PERMANOVA and betadisper were adjusted using the false discovery rate (FDR) method. All these analyses were implemented using the vegan package (v2.7-1) in R (v4.5.1).

Mantel tests evaluating the relationships between different subcommunities and environmental variables were implemented using the linkET package (v0.1.0) in R (v4.5.1). Additionally, redundancy analysis (RDA) was performed using the vegan package (v2.7-1) to link ASV subcommunities to environmental factors along temporal and spatial distance gradients. To control for multiple comparisons, *P*-values derived from both the Mantel tests and RDA were adjusted using the FDR method. Differentially abundant biomarkers were identified via linear discriminant analysis effect size (LEfSe, v1.0.8) with an LDA score threshold of 3.5.

Correlation analysis between pairwise Bray-Curtis distances and geographic distances was performed using the tidyverse (v2.0.0) and boot (v1.3-30) packages in R software (v4.5.1). Resampling (resampling = 2,000) was used to compute 95% confidence intervals (CIs) for Pearson correlation coefficients, and the FDR method correction was applied to adjust the resulting *P*-values to formally test the differences between groups.

A neutral community model (NCM) was further fitted to disentangle the relative contributions of stochastic and deterministic processes to community assembly. For subcommunities where species richness is too low or data is excessively sparse, resulting in the failure of non-linear fitting to converge, they were excluded from the NCM analysis.

To evaluate the predictive utility of bacterial and fungal communities for identifying soil origins across the five sampling locations, RF classification models were constructed using the scikit-learn package (v1.3.0) in Python (v3.10.12). Data preprocessing, matrix transformations, and relative abundance normalizations were executed utilizing the pandas package (v2.0.3). To rigorously prevent temporal information leakage, a leave-one-date-out nested cross-validation (LODO-NCV) strategy was implemented. In the outer loop, the dataset was partitioned by sampling date: in each iteration, data from four dates (60 samples) were used for training, while the remaining date (15 samples) was completely withheld as an independent test set. Within the inner loop, time-grouped cross-validation was performed exclusively on the training samples for hyperparameter tuning. To ensure the stability of performance metrics and mitigate fluctuations caused by limited sample sizes, the entire LODO-NCV procedure was repeated 10 times with different random seeds. Model performance was evaluated using accuracy, Cohen's kappa, and recall, and the results are reported as means with 95% confidence intervals (CIs). Statistical comparisons of model performance were assessed via paired *t*-tests or Wilcoxon signed-rank tests depending on data normality, implemented using the scipy (v1.11.2) package.

Furthermore, to identify the core predictive taxa, the top 20 most important bacterial and fungal features were extracted from the optimized models based on the Mean Decrease in Gini impurity metric. All performance and feature importance visualizations were generated using the matplotlib (v3.6.3) package.

## 3 Results

### 3.1 Alpha diversity of bacterial and fungal communities

Alpha rarefaction curves for both bacteria and fungi demonstrated that, with increasing sequencing depth, the curves for all samples plateaued before reaching the rarefaction threshold (Supplementary Figure 1). Community richness, Shannon index, and Pielou's evenness were calculated to evaluate species richness and evenness across spatial (sampling site) and temporal (sampling time) gradients (Supplementary Figure 2). Among sampling sites, only bacterial Pielou's evenness differed significantly (*P* < 0.05); other alpha diversity indices didn't show significant spatial variation for either bacteria or fungi. Across sampling times, none of the alpha diversity indices exhibited significant differences in bacterial or fungal communities.

### 3.2 Microbial community composition of soil samples in different sites

**Figure 1:**
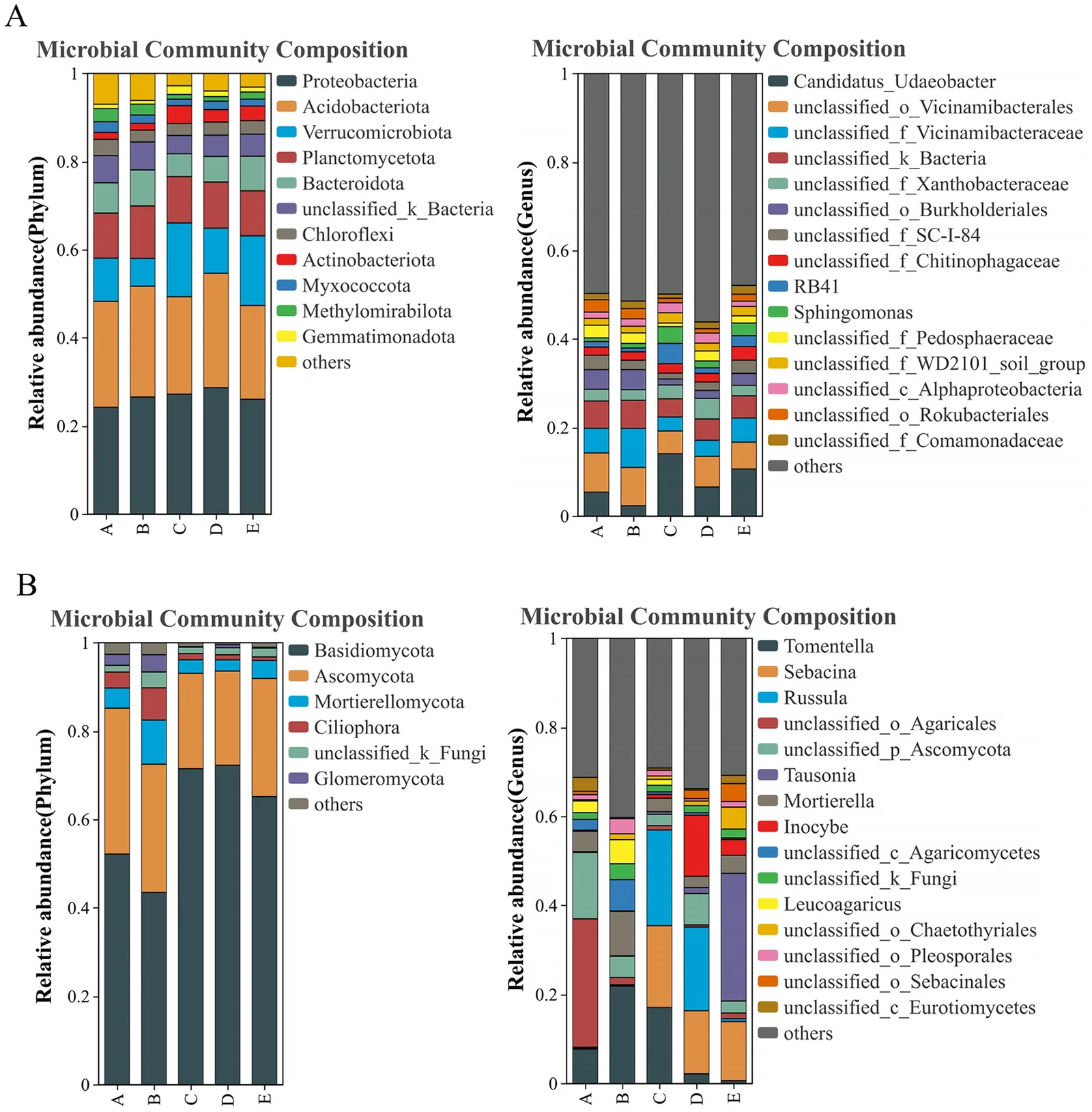
Microbial community composition of bacteria **(A)** and fungi **(B)** at the phylum and genus levels under location grouping.

The microbial community composition of soil samples was characterized at both the phylum and genus levels (Figure 1). At the phylum level, overall community composition showed no apparent variation among sampling sites; bacteria were dominated by *Proteobacteria* and *Acidobacteriota*, while fungi were predominantly composed of *Basidiomycota* and *Ascomycota*. At the genus level, bacterial communities were largely characterized by *Candidatus Udaeobacter, unclassified _o_Vicinamibacterales*, and *unclassified_f_Vicinamibacteraceae*, with unclassified taxa constituting the majority (Figure 1A). In contrast, fungal genera exhibited pronounced spatial heterogeneity (Figure 1B): Site A was mainly characterized by *unclassified_o_Agaricales*; Site B by *Tomentella, Mortierella*, and *unclassified Agaricomycetes*; Site C by *Tomentella, Sebacina*, and *Russula*; Site D by *Sebacina, Russula*, and *Inocybe*; and Site E by *Sebacina* and *Tausonia*. Bacterial and fungal community composition showed no apparent temporal shifts across sampling times (Supplementary Figure 3).

To further elucidate spatial structuring, hierarchical clustering based on the relative abundance profiles at the genus level was performed for both bacteria and fungi (Supplementary Figure 4). The results revealed that the majority of samples clustered clearly according to their sampling sites, regardless of whether the analysis was based on bacterial or fungal abundance profiles.

### 3.3 Analysis of spatiotemporal factors associated with community structure

**Figure 2:**
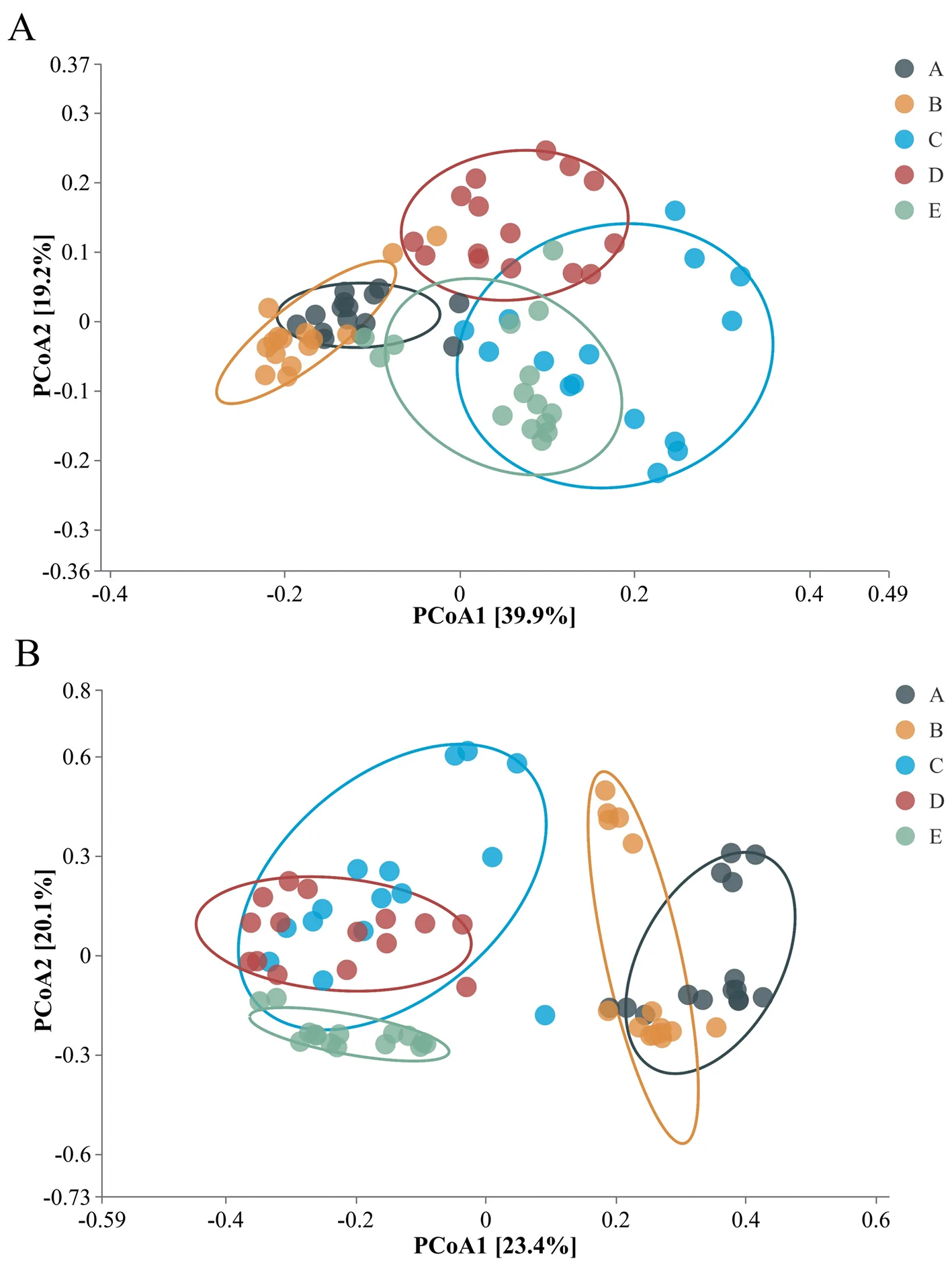
PCoA of samples from different sampling sites based on bacterial **(A)** and fungal **(B)** communities.

Bray-Curtis dissimilarities of soil samples was estimated by genus-level relative abundances of bacterial and fungi, respectively. PCoA of soil samples was conducted by their pairwise Bray-Curtis dissimilarities. As shown in Figure 2, most samples clustered by sampling sites based on either bacterial or fungal communities. In addition, compared with the bacterial PCoA, samples in the fungal PCoA exhibited a stronger spatial clustering tendency and were sequentially separated in accordance with sampling locations. Nevertheless, when samples were grouped by sampling time, their distributions were relatively scattered for both bacterial and fungal datasets, with no distinct temporal clustering patterns observed (Supplementary Figure 5).

We also performed PERMANOVA and tests of multivariate dispersions to quantify the explanatory power of spatial and temporal factors on community variation (Table 2 and Supplementary Table 2). The results demonstrated that sampling location was extremely significantly associated with the structural variation of both bacterial (*R*^2^ = 0.37, *P_FDR* < 0.001) and fungal communities (*R*^2^ = 0.45, *P_FDR* < 0.001). Notably, the betadisper analysis confirmed that fungal community dispersions were homogeneous across all sampling sites (*P_FDR* = 0.077), indicating that the observed differences were robustly linked to spatial location rather than within-group dispersion heterogeneity. For bacterial communities, although marginal global dispersion heterogeneity was detected (*P_FDR* = 0.029), the majority of pairwise spatial comparisons exhibited homogeneous dispersions (Table 2). In contrast, temporal factors exhibited no significant correlation with the community structural variation of bacteria or fungi within the observation window (*P* > 0.05; Supplementary Table 2).

**Table 2:** PERMANOVA and betadisper analyses of soil samples from different sampling sites by bacterial and fungal communities.

Groups	Bacteria				Fungi			
	*F*	*R^2^*	*P_fdr*	*PERMDISP _P_fdr*	*F*	*R^2^*	*P_fdr*	*PERMDISP _P_fdr*
A vs. B	10.27	0.27	<0.001	0.985	8.77	0.24	<0.001	0.46
A vs. C	16.39	0.37	<0.001	0.01	15.60	0.36	<0.001	0.551
A vs. D	10.40	0.27	<0.001	0.597	18.70	0.40	<0.001	0.741
A vs. E	7.75	0.22	<0.001	0.025	25.50	0.48	<0.001	0.435
B vs. C	13.65	0.33	<0.001	0.105	10.30	0.27	<0.001	0.797
B vs. D	10.08	0.26	<0.001	0.706	15.40	0.36	<0.001	0.551
B vs. E	5.72	0.17	<0.001	0.105	18.70	0.40	<0.001	0.06
C vs. D	12.13	0.30	<0.001	0.105	4.66	0.14	<0.001	0.669
C vs. E	9.49	0.25	<0.001	0.985	16.20	0.37	<0.001	0.065
D vs. E	7.38	0.21	<0.001	0.105	16.10	0.37	<0.001	0.087
All_groups	10.29	0.37	<0.001	0.029	14.50	0.45	<0.001	0.077

**Figure 3:**
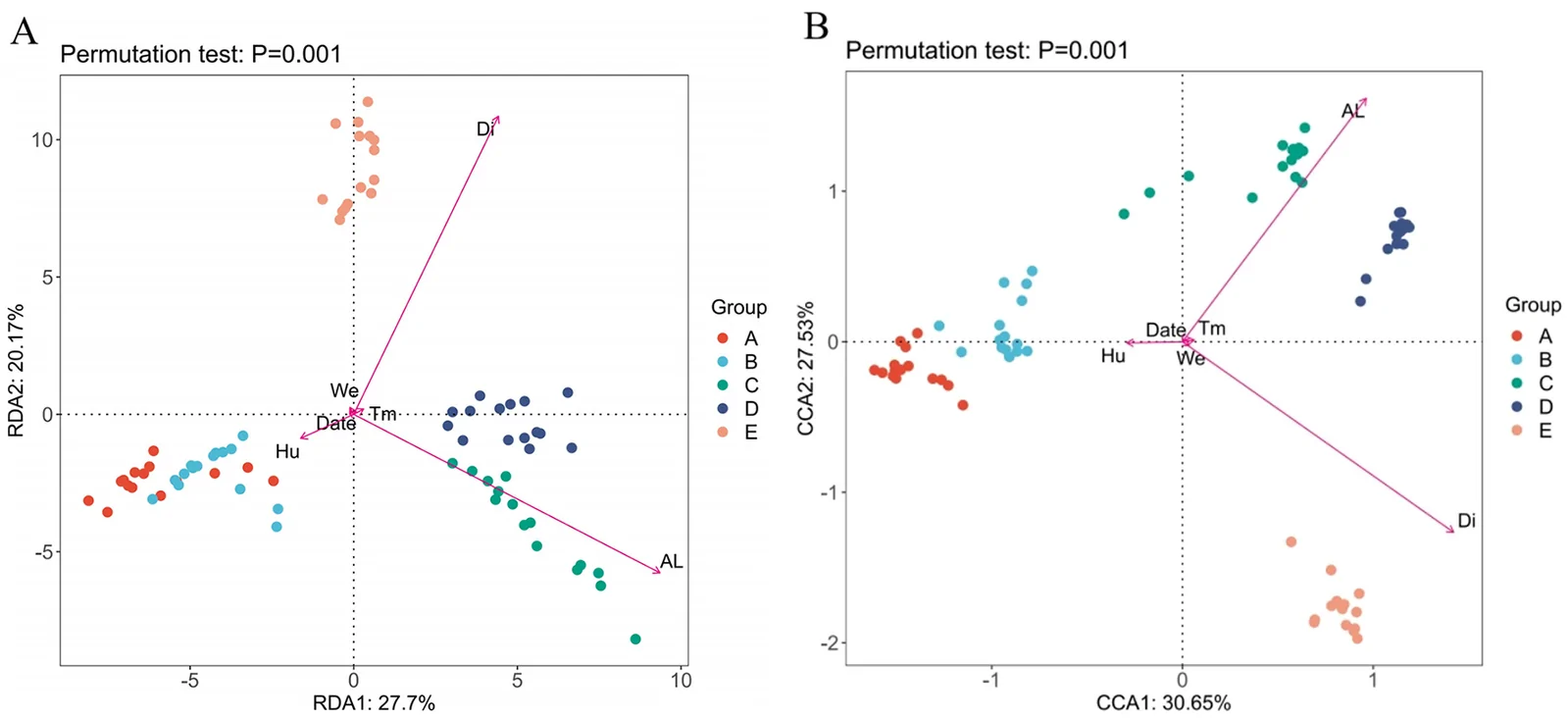
RDA of environmental factors and soil samples by bacterial **(A)** and fungal **(B)** communities under location grouping.

RDA was also performed to evaluate the effects of environmental variables on sample distribution (Figure 3). The results indicated that two environmental factors, geographic distance and altitude, were significantly associated with community structure, regardless of whether samples were grouped by sampling site (Figure 3) or sampling time (Supplementary Figure 6).

### 3.4 Environmental factors associated with microbial subcommunity structure

**Figure 4:**
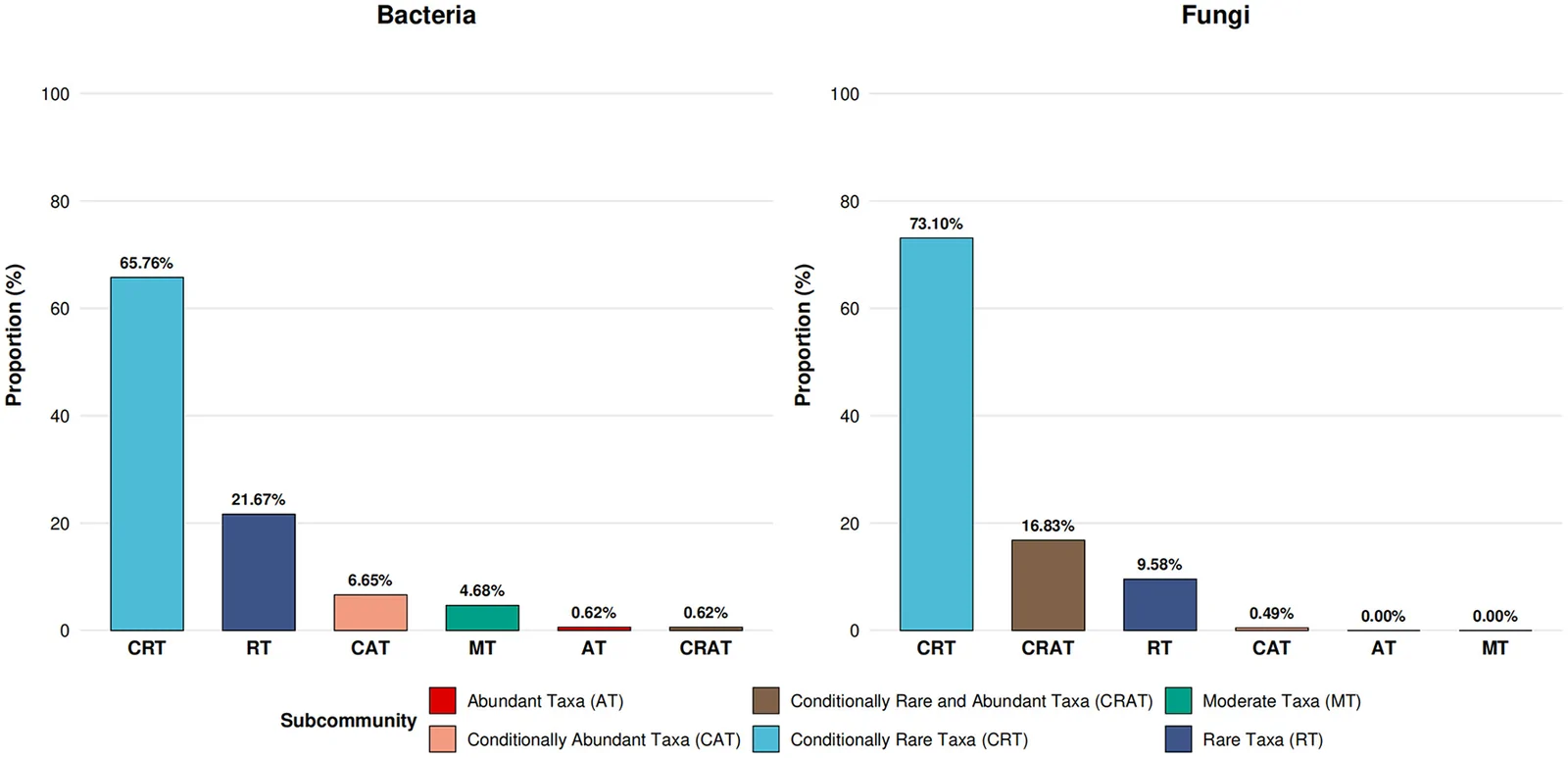
Bar charts of bacterial and fungal subcommunities categorized by the relative abundance of ASVs in soil samples.

To elucidate how environmental factors correlate with microbial subcommunity structure, ASVs were classified into six subcommunities based on their relative abundance and occurrence frequency across samples (Chen et al., 2017): abundant taxa (AT; ≥ 1% in all samples), rare taxa (RT; <0.01% in all samples), moderate taxa (MT; 0.01–1% in all samples), conditionally rare taxa (CRT; <1% in all samples but <0.01% in some samples), conditionally abundant taxa (CAT; ≥ 0.01% in all samples but ≥ 1% in some samples), and conditionally rare and abundant taxa (CRAT; <0.01% in some samples and ≥ 1% in some samples). Both bacterial and fungal communities were dominated by the CRT subcommunity (Figure 4).

**Figure 5:**
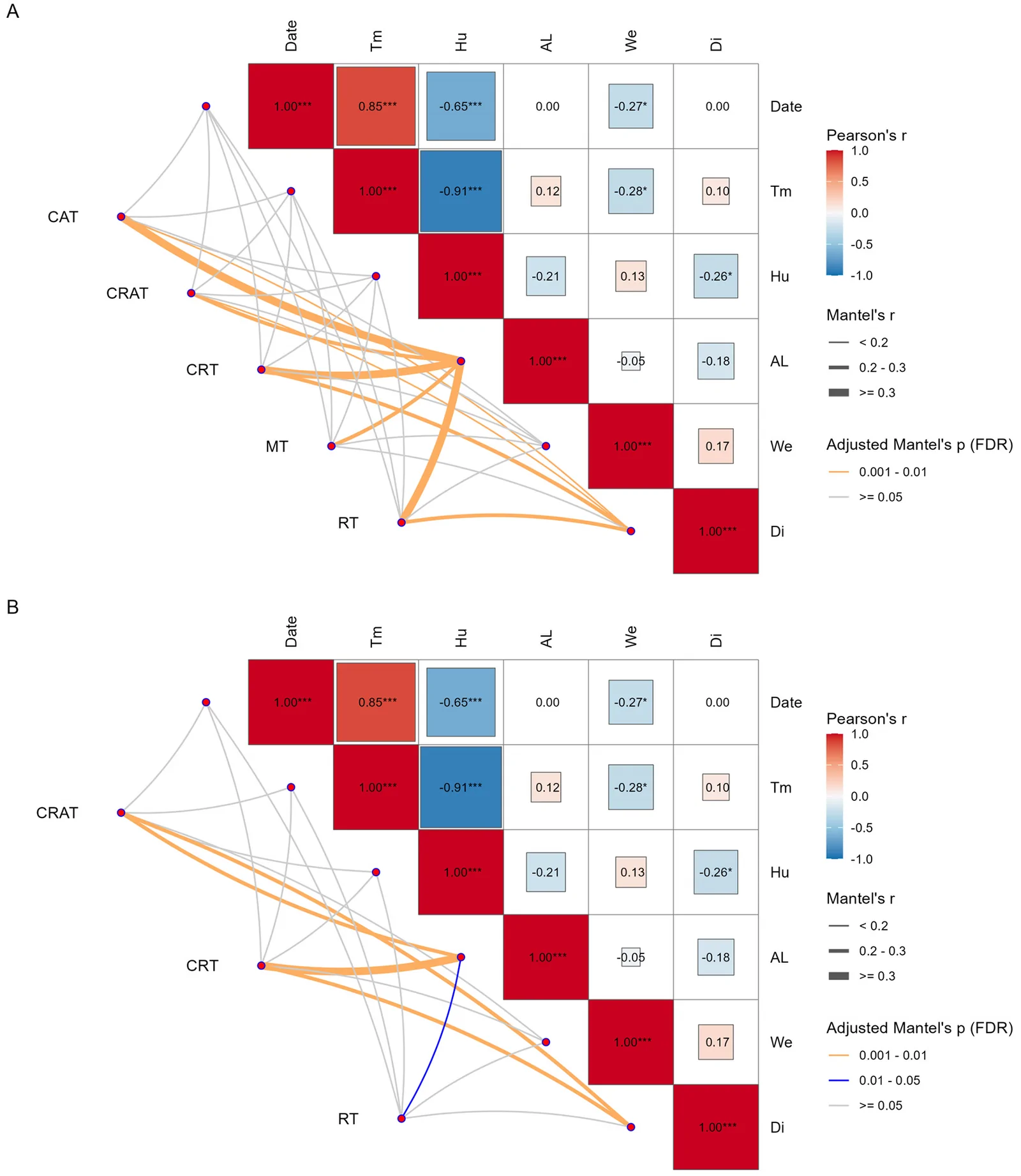
Mantel analysis of environmental factors and bacterial subcommunities **(A)** and fungal subcommunities **(B)**.

**Figure 6:**
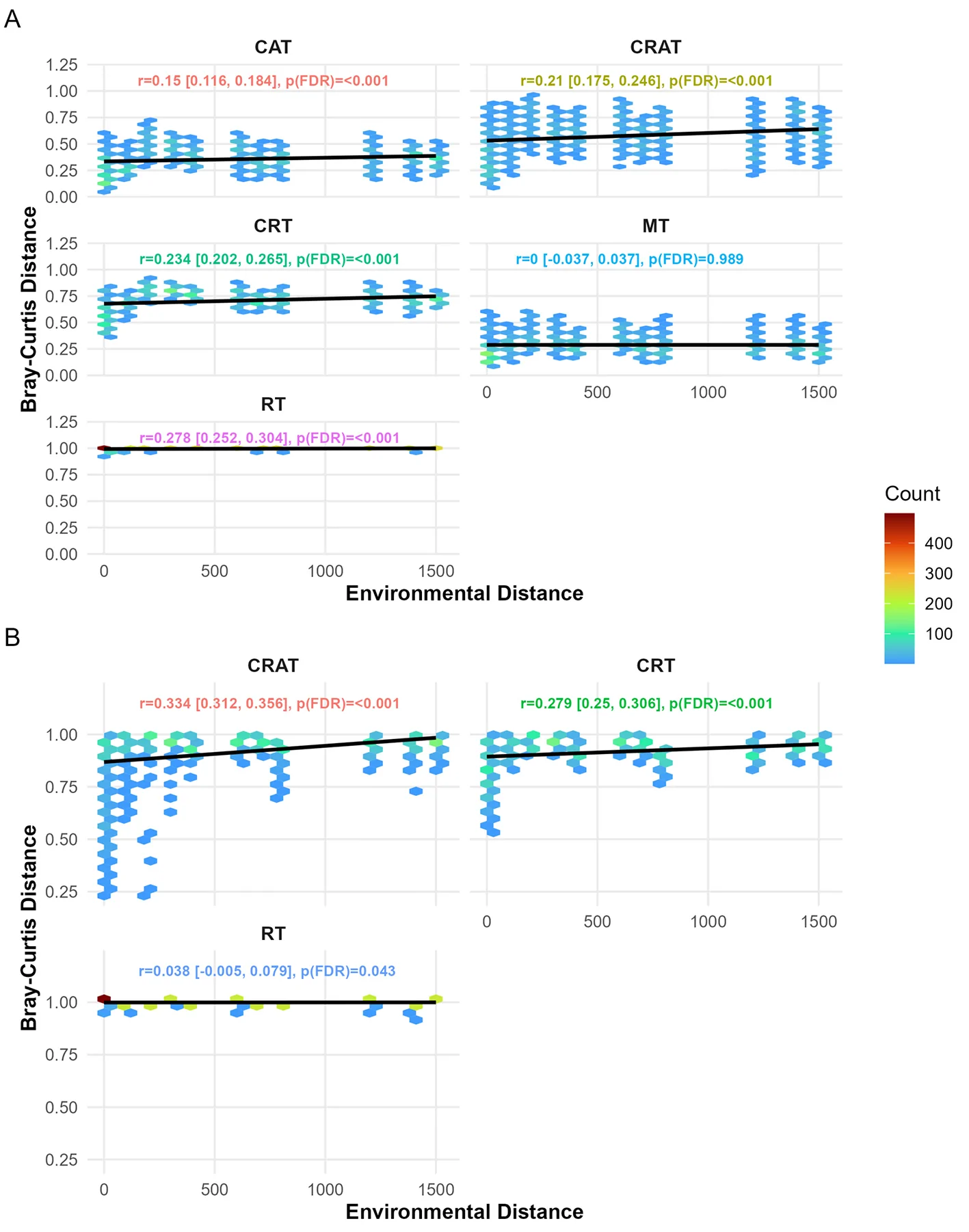
Distance-decay patterns of Bray-Curtis distances and environmental distances based on bacterial **(A)** and fungal **(B)** subcommunities.

Regardless of the community type analyzed, RDA revealed that altitude and geographic distance acted as the primary spatial proxies for soil samples (Supplementary Figures 7, 8). Mantel tests further confirmed that altitude and geographic distance were strongly correlated with bacterial subcommunities (CRT, CRAT and CAT) and fungi subcommunities (CRT and CRAT) (*P_*FDR <0.05) (Figure 5). Distance-decay relationship analysis revealed that, with the exception of the bacterial MT subcommunity, the Bray-Curtis dissimilarities of all remaining subcommunities increased significantly with increasing geographic distance (*P_*FDR <0.05) (Figure 6). Notably, resampling analysis demonstrated that the Pearson correlation coefficient (r) of the fungal CRT subcommunity [*r* = 0.279, 95% CI: (0.25, 0.306)] was higher than that of the bacterial CRT subcommunity [*r* = 0.234, 95% CI: (0.202, 0.265)], indicating a notable spatial dependence in fungal communities within the habitat range of this study. It should be noted that the differences in correlation coefficients between the fungal and bacterial sub-communities were assessed based on visual observation of their confidence intervals, and no formal statistical tests were conducted to directly compare these differences.

### 3.5 Neutral community model analysis

**Figure 7:**
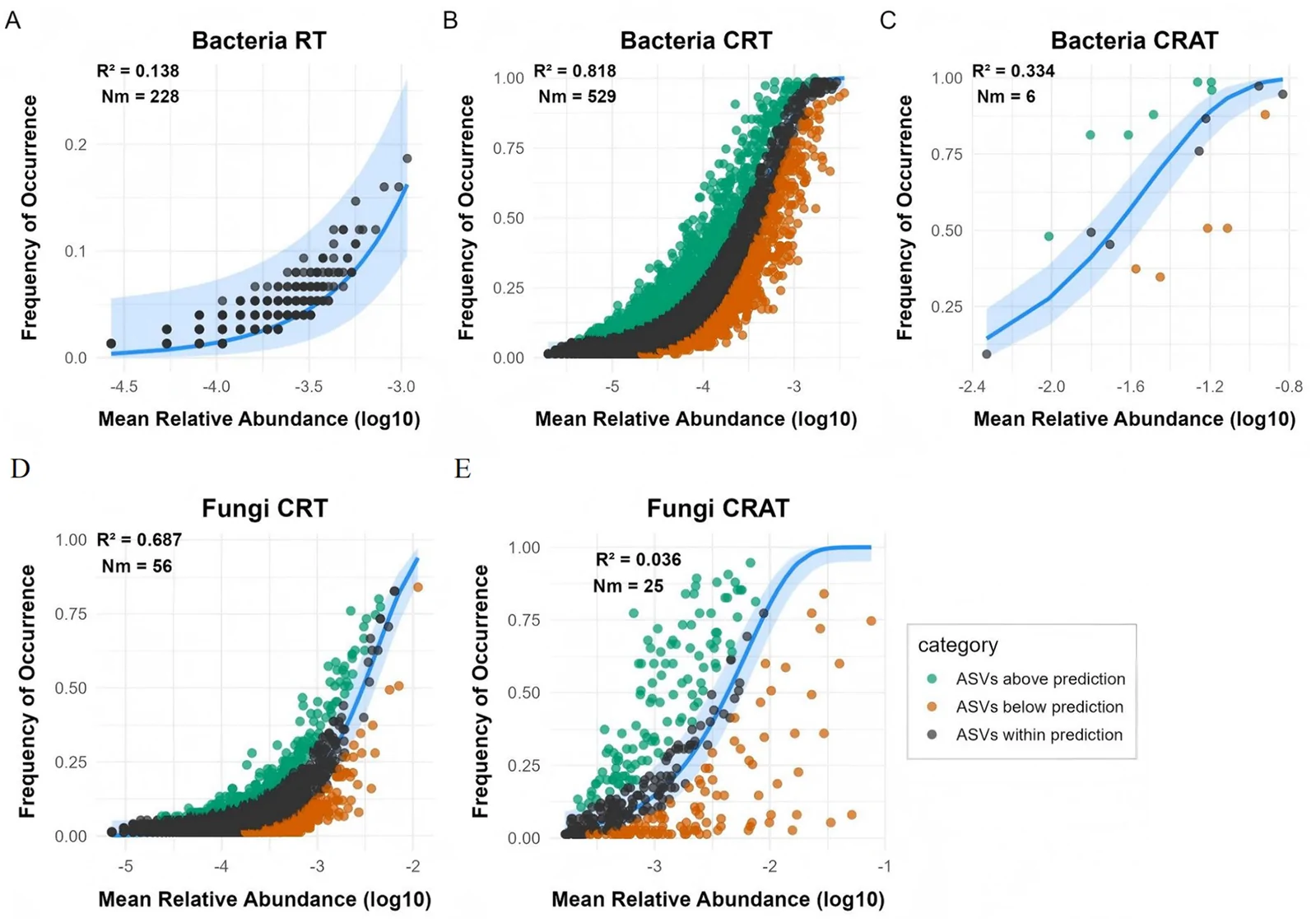
Neutral community models of bacterial and fungal subcommunities. **(A)** Bacteria RT; **(B)** Bacteria CRT; **(C)** Bacteria CRAT; **(D)** Fungi CRT; **(E)** Fungi CRAT.

Neutral community model (NCM) analysis was applied to assess the relative contributions of stochastic and deterministic processes to community assembly. We conducted NCM analyses on the sub-communities of bacteria and fungi (Figure 7). The results showed that the CRT sub-communities exhibited the highest model goodness-of-fit for both bacterial and fungal groups. Specifically, the neutral fit for the bacterial CRT (*R*^2^ = 0.818, Nm = 529) subcommunity was stronger than that of the fungal CRT (*R*^2^ = 0.687, Nm = 56) subcommunity, though both demonstrated robust signals of neutral processes. Overall, all analyzed fungal subcommunities displayed lower NCM goodness-of-fit values and Nm values than corresponding bacterial subcommunities, suggesting that fungal communities were relatively more strongly governed by deterministic processes like environmental filtering and dispersal limitation than bacterial communities.

### 3.6 Screening of geographically characteristic biomarkers and construction of prediction models

Based on the above analyses, geographic distance and altitude constituted the primary factors associated with bacterial and fungal community structures. Accordingly, we further screened bacterial and fungal biomarkers that exhibited significantly differential distributions across sampling sites in subsequent analyses. LEfSe identified 116 bacterial and 124 fungal biomarkers with significantly differential distributions across different sampling sites at the threshold of LDA > 3.5 (Supplementary Figure 9; Supplementary Tables S3, S4).

**Figure 8:**
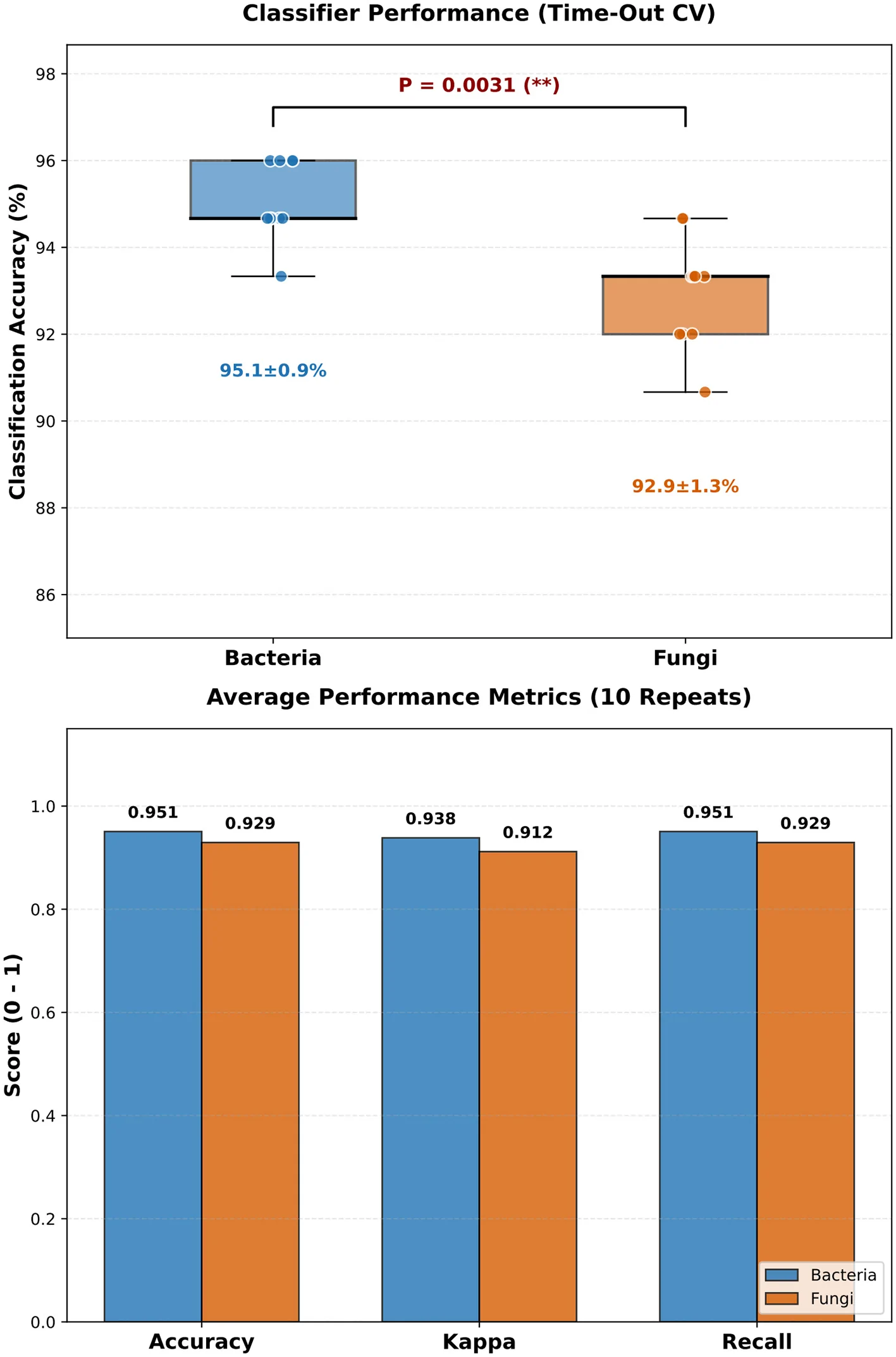
Predictive performance of the random forest (RF) models for soil geographic provenance based on bacterial and fungal communities.

Subsequently, machine learning predictive models were built via the RF algorithm based on the relative abundances of bacterial and fungal taxa separately, to explore the predictive capacity of bacterial and fungal taxa for the geographic origin of soil samples at a small spatial scale. To prevent temporal information leakage, a leave-one-date-out nested cross-validation strategy with 10 repeats was implemented. The classification results of the established models demonstrated that after isolating the confounding effects of temporal leakage, both models exhibited highly robust predictive performance. Specifically, the bacterial RF model achieved an average accuracy of 95.1 ± 0.9%, alongside a mean Cohen's kappa of 0.938 and a recall of 0.951. The fungal RF model yielded an average accuracy of 92.9 ± 1.3%, with a mean kappa of 0.912 and a recall of 0.929 (Figure 8). Furthermore, to identify the core predictive taxa, the top 20 most important bacterial and fungal features were extracted based on the Mean Decrease Gini metric (Supplementary Figure 10), implying that these core species may exhibit strong spatial discriminatory power among the sampling sites.

## 4 Discussion

A central finding of this study is that, during the continuous 14-day summer sampling period, spatial heterogeneity in soil environments was significantly more associated with microbial community structure than temporal fluctuation. This conclusion was also supported by PERMANOVA results, which revealed a highly significant effect of geographic distance (*P* < 0.001) but no significant effect of sampling time. While previous studies have demonstrated the primacy of spatial over temporal effects in soil microbial communities at broad geographic scales, our study extends this critical finding to the fine-scale spatial and short temporal scales most directly relevant to forensic investigations. Liu et al. identified geographic distance as the primary driver of soil bacterial community assembly, demonstrating a pronounced distance-decay relationship (Liu et al., 2023). Similarly, Hannula et al. showed that legacy effects in soil microbial networks, particularly among fungal endophytes, can persist for months despite environmental change (Hannula et al., 2021). The temporal monitoring conducted by Fox et al. also demonstrated that geographic soil systems explained far more variation in community composition than temporal fluctuations (Fox et al., 2022). The relative temporal stability provides critical empirical support for the “lagged sampling” scenarios frequently encountered in forensic investigations, indicating that soil trace evidence collected within a brief post-incident window (e.g., up to 2 weeks) could retain robust microbial geographic signatures resistant to short-term environmental perturbation.

Within the spatiotemporal scope of this study, both bacterial and fungal communities proved to be highly effective geographic provenance biomarkers. While fungal communities exhibited stronger spatial dependence and environmental filtering, the bacterial RF model slightly outperformed the fungal counterpart in closed-set prediction tasks. Distance-decay analyses revealed significant correlation coefficients between Bray-Curtis distances and environmental distances for both bacterial and fungal subcommunities (e.g., CRT), indicating clear spatial dependence in both groups. NCM analysis further elucidated the underlying assembly mechanisms: lower goodness-of-fit in fungal communities indicated that their assembly was predominantly governed by deterministic processes, including environmental filtering and dispersal limitation. This strong environmental dependency renders fungal communities highly sensitive to fine-scale variation in microtopography and local physicochemical conditions. Despite the stronger spatial heterogeneity of fungi, RF models achieved slightly higher predictive accuracy using bacterial biomarkers than fungi markers (95.1% vs 92.9%), validating the utility of both as core biological tracers while highlighting the robustness of bacterial fingerprints in small-scale closed-set predictions. Interestingly, the bacterial community harbored numerous unclassified genera (e.g., unclassified_o_Vicinamibacterales). Although the ecological roles of these taxa—often referred to as “microbial dark matter”—remain poorly characterized (Zha et al., 2022), our findings demonstrate that within the short-term and micro-scale constraints, such taxonomic ambiguity did not compromise their forensic utility. On the contrary, the immense richness of the bacterial microbiome provide a highly dense and robust quantitative fingerprint, enabling the RF algorithm to achieve slightly superior classification accuracy (95.1%). Even so, future forensic soil research should integrate metagenomic approaches to achieve deeper taxonomic resolution and establish standardized regional reference databases to provide essential benchmarks for microbial geospatial sourcing.

Despite these promising findings, several limitations warrant consideration before these results can be generalized to routine forensic casework. First, soil physicochemical characterization (Like pH, organic matter, and moisture) was not conducted, as the available sample material was exhausted during DNA extraction. Given that soil pH is widely recognized as one of the most robust predictors of bacterial community assembly at global scales, its absence limited deeper mechanistic interpretation of the observed geographic signals. Second, we acknowledge that the strong explanatory power of geographic distance and elevation revealed by RDA for fungal communities is confounded with shifts in vegetation composition. As the five sampling sites co-vary in distance, elevation, and dominant tree species, it is not possible to statistically disentangle the pure effect of geographic distance from habitat filtering. Therefore, the observed spatial patterns likely reflect a combined signal of dispersal limitation and environmental selection driven by host tree identity. This pattern is notably driven by the turnover of ectomycorrhizal taxa (e.g., *Tomentella* and *Russula*) accompanying the replacement of host tree species. Without concurrent vegetation and edaphic data, fully disentangling these intertwined effects remains challenging. Third, the temporal stability observed in this study is confined to a continuous 14-day summer sampling period and cannot capture potential community shifts driven by seasonal precipitation or temperature fluctuations. Future investigations encompassing complete seasonal cycles and extended time series are needed to comprehensively evaluate long-term temporal dynamics and their potential influence on forensic tracing accuracy. Furthermore, as a proof-of-concept conducted within a single forest environment, the geographic transferability of these biomarkers across diverse forest types, climate zones, and macro-scale soil gradients remains to be established through broader cross-regional validation. Last, the current RF framework is inherently a closed-set classification model restricted to five predefined micro-scale locations. Extending forensic soil provenance to unknown samples across broader geographic scales will require the establishment of comprehensive regional baseline databases alongside the development of open-set recognition algorithms (Zhang, 2025).

Beyond these methodological constraints, practical forensic applications pose additional challenges. In real casework, soil residues attached to suspects' shoe soles, tires, or clothing are typically present in trace amounts and frequently mixed with materials from multiple contact sites, including residences, crime scenes, and transit locations (Robinson et al., 2020; Demaneche et al., 2017). Such mixed-source contamination can produce microbial profiles markedly divergent from native community signatures, posing severe obstacles to conventional comparative analyses and source-tracing models. Developing bioinformatic algorithms to efficiently deconvolve core characteristic communities from complex mixed trace evidence thus represents a critical bottleneck for future forensic microbiology research. From a technical standpoint, while targeted amplicon sequencing (16S rRNA and ITS) delivers high sensitivity for low-biomass samples, it captures only specific hypervariable regions, lacking strain-level taxonomic resolution and comprehensive functional metabolic profiles (Bhattacharya et al., 2022; Zhang et al., 2023). Future studies integrating metagenomic or long-read sequencing approaches are warranted to achieve finer taxonomic discrimination. Nevertheless, this study provided a solid theoretical foundation and proof-of-concept for the microbial characterization of short-term forensic soil evidence, paving the way for future forensic soil microbiome research.

## 5 Conclusions

Using high-throughput sequencing technology, this study systematically evaluated the associations between temporal dynamics and spatial distance on soil bacterial and fungal communities. We verified that the internal structure of soil microbial communities did not undergo significant succession within the 14-day observation window. Compared with bacteria, soil fungal communities were subjected to stronger environmental filtering and dispersal limitation, exhibiting more pronounced distance-decay patterns and higher spatial heterogeneity. Machine learning-based closed-set classification revealed that both microbial groups possess extremely high discriminatory capacity for distinguishing among the predefined sampling sites. Accordingly, both bacterial and fungal communities can serve as highly effective biological tracers for forensic soil provenance.

## Data Availability

The raw sequence data reported in this paper have been deposited in the Genome Sequence Archive in National Genomics Data Center, China National Center for Bioinformation/Beijing Institute of Genomics, Chinese Academy of Sciences (GSA: CRA047197) that are publicly accessible at https://ngdc.cncb.ac.cn/gsa.
