# Supplementary material for: Spatiotemporal dynamics of soil bacterial and fungal communities at a small scale
Source: Front Microbiol. 2026 Sep 11;17:1924980. doi: 10.3389/fmicb.2026.1924980 (PMC13612575; doi:10.3389/fmicb.2026.1924980)
Supplement: Supplementary file 1 [file Table_1.docx]

**Supplementary Figures**

**Fig. S1.** Rarefaction curves for bacteria (A) and fungi (B) across samples.

**Fig. S2.** Alpha diversity indices of bacterial and fungal communities across sampling sites (A) and time points (B).

**Fig. S3.** Microbial community composition of bacteria (A) and fungi (B) at the phylum and genus levels in different sampling time.

**Fig. S4.** Dendrogram of samples based on the genus abundance profiles of bacteria (A) and fungi (B).

**Fig. S5.** PCoA of samples from different sampling time based on bacterial (A) and fungal (B) communities.

**Fig. S6.** RDA of environmental factors and soil samples by bacterial (A) and fungal (B) communities under sampling time grouping.

**Fig. S7.** RDA of environmental factors and soil samples based on bacterial subcommunities (A–C) and fungal subcommunities (D–F) across spatial gradients.

**Fig. S8.** RDA of environmental factors and soil samples based on bacterial subcommunities (A–C) and fungal subcommunities (D–F) across different sampling time.

**Fig. S9.** Linear discriminant analysis effect size analysis identifying differential bacterial (left) and fungal (right) taxa among sampling sites.

**Fig. S10.** The top 20 most predictive taxa when classifying soil geographic provenance based on bacterial and fungal communities in the samples
